# Foreign Summary

**Published:** 1839-11-30

**Authors:** 


### FOREIGN SUMMARY.

### Velpeau's Clinical Lectures on Ophthalmia. No. XI.

#### SEQUELÆ AND COMPLICATIONS OF KERATITIS.

*Ulcers of the Cornea.—Different modes of treatment.—Necessity of first curing the inflammation.—The cicatrix liable to be rendered opaque by preparations of lead.—Remarks on cauterization.—Necessity of avoiding this when there is acute inflammation.—Delicacy of the operation.—Excision of the injected vessels.—Mode of effecting this.—Perforation and fistulæ of the cornea.*

Two causes have hitherto contributed to prevent the treatment of ulcers of the cornea being properly understood; first, their having always been considered apart from the inflammatory affection which has caused them, or by which they are accompanied; and secondly, the modifications which they present not having been generally recognised. This manner of viewing ulceration of the cornea has given rise to ideas respecting the treatment of such ulcers which ought not to pass unrefuted. Thus many practitioners consider it advisable to treat these lesions by direct cauterization, or by the excision of the vascular filaments which are distributed to them. Such measures are sometimes useful, it is true, but not when employed in every kind of ulcer, and in every stage of the affection. Those who indiscriminately advise this plan of treatment, seem not to be aware that an ulcer nearly always arises from keratitis, and that, consequently, if we wish to cure the ulcer, we must first cure the inflammatory affection of the cornea. There are, however, some surgeons, such as Mr. Lawrence, who disapprove of cauterization and excision during the acute period of the inflammation, and recommend antiphlogistic measures to be adopted. Scarpa may be named as one of those who place the greatest reliance on excision, &c.

Ulcers of the cornea often disappear, even when abandoned to nature, and oftener still under the influence of the treatment calculated to remove the keratitis. As, however, they do not always yield so easily, it is necessary that you should be acquainted with those remedial agents which are more especially directed against them.

When ulceration of the cornea is recent, and accompanied by keratitis, the presence of which is indicated by the vascularity of the sclerotica and conjunctiva, the first thing to be done is to cure the keratitis, as by doing so you generally cure the ulcer also. Should it continue, you may employ calomel in powder, a solution of the sulphate of zinc or of the nitrate of silver. This last preparation is decidedly the most efficacious, and seldom fails. Sometimes, however, even this remedy proves ineffectual; in which case you must have recourse to cauterization, or to the excision of the superficial varicose vessels, which are sometimes found around the ulcer.

The same plan of treatment is generally successful when directed against the plastic ulcer. In treating this kind of ulcer, it is well to avoid the various preparations of lead, as I have found, by experience, that small particles of that substance employed may be deposited at the bottom of the ulcer, and thus render the cicatrix more opaque. Here, again, if other measures fail, cauterization or excision of the injected vessels may be resorted to. It is, however, useless to cauterize as long as the ulcer is covered by a layer of lymph, the surface of the cornea being then protected in the same manner as if it were covered by an eschar.

The ulcer requires no special treatment as long as the inflammation of the cornea continues.—When that has abated, the astringent collyria should be employed, and if they do not succeed, as is frequently the case, recourse must be had to cauterization with the nitrate of silver. It is, indeed, in this species of ulcer that cauterization is the most efficacious. The cure, however, is seldom radical; there nearly always remains, when the ulcer is deep, a speck which impedes the functions of sight more or less, according to the position which it occupies on the cornea. Excision of the injected vessels evidently cannot be performed in these cases, as it is not by the vessels of the conjunctiva, but by those of the sclerotica, that the partial vascularity of the cornea is kept up.

When the tissue of the cornea has been entirely destroyed, and the membrane of the aqueous humour appears at the bottom of the ulcer, topical remedies have but little influence over the progress of the lesion; general measures, and especially blood-letting, being indicated. I have sometimes, however, in these cases, derived benefit from a collyrium composed of one grain of the sulphate of zinc to an ounce of water with some astringent mucilage. If the internal lamellæ of the cornea protrude, cauterization may be resorted to, but only with the greatest caution. Blisters applied over the eyelids are also occasionally useful. The remarks which I have made on the treatment of the first three species of ulcers will also apply to that of the two latter, as long as they are accompanied by acute inflammation. But when the inflammation has subsided, the treatment is no longer the same, cauterization of the ulcer being scarcely ever attended with beneficial results, and that of the incised ulcer being absolutely prejudicial. The non-success of cauterization, when directed against the incised ulcer, may be easily explained. The incised ulcer forming, as it were, a deep fissure, it is impossible to act on its entire surface with the solid nitrate of silver; and when cauterization is resorted to in any form of ulceration of the cornea, unless the entire ulcerated surface be cauterized, the operation merely tends to increase the intensity of the disease. The ulcer being also nearly always situated at the circumference of the cornea, the conjunctiva is generally cauterized at the same time, and its inflammation thereby increased.

To resume: cauterization ought not to be employed when there is acute inflammation of the cornea co-existing, unless it be in those cases in which the ulcer has given rise to the inflammation, or in those in which vessels are seen arising, as it were, from the ulcerated surface. When the inflammation has subsided, cauterization is, on the contrary, in some species of ulcers, the best remedy that can be used, as it destroys the extreme sensibility which always exists when the tissue of the cornea is denuded. For such an effect, however, to be produced, the cauterization must be carefully executed, and this is attended with some difficulty. The photophobia being generally very intense, the patient forcibly contracts his eyelids, so that it is by no means easy to keep them open, or to maintain the eye in the same position. Now when you consider that the slightest motion may cause you to cauterize another portion of the cornea instead of the ulcer, and that, on the other hand, it is indispensable that the entire ulcerated surface should be cauterized, you must certainly agree with me that the operation, slight as it appears, is one which requires great nicety, to be properly performed. When cauterization is resorted to, the entire ulcerated cavity must be touched with a cone of lunar caustic, rounded at the extremity. This must be done very lightly when the ulcer is deep, in order that the remaining layers of the cornea may not be destroyed. Some tepid water must then be poured over the eye, before the patient is allowed to close his eyelids, to prevent the caustic acting on the surrounding tissue. When the cauterization has been effectually performed, the severe pain which the patient at first feels soon subsides, and he then suffers much less than he did before the cauterization. On the third or fourth day the photophobia and epiphora generally return, owing to the falling of the small eschar which is formed, and it is sometimes necessary to repeat the cauterization two or three times.

The only plan of treatment, besides cauterization, which I have mentioned as being specially directed against ulcers of the cornea, is the excision of the injected vessels. Excision has been often resorted to when it ought not to have been employed, and having then proved rather detrimental than otherwise, has been entirely rejected by most practitioners. Though of little or no use when the vascularization of the cornea is supplied by the vessels of the sclerotica, it may be attended with beneficial results in superficial keratitis, accompanied by inflammation of the conjunctiva, and by injection of the superficial vessels of the inflamed membrane. The vessels must be seized with a small pair of forceps, and are then excised with ease. Sometimes, when the cornea is covered with small ulcers, the principal vascular filaments are given off from the conjunctiva, in which case their excision is indicated. Some authors have proposed the excision of a circular portion of the conjunctiva. This proposal, though favourably received by a few practitioners, has not met with the same reception from others. The remarks I have just made will equally well apply in this instance. If the keratitis is kept up by the conjunctival vessels, excision of a portion of the conjunctiva is likely to prove efficacious; but if it is kept up by the deep-seated vascular layer, the operation cannot be of much avail. You must also bear in mind that excision of a part of the vascular mucous membrane may, as I have already told you, be followed by disagreeable consequences.

Ophthalmologists have advised that the surface of the ulcer be scraped or excised, to prevent the formation of an albugo or a leucoma. This plan of treatment is evidently applicable to those ulcers only on which there exists a layer of coagulable lymph, and even then I can hardly say how far it would prove advantageous. Such an operation would also require such great manual dexterity, that I do not think many surgeons of our own times will feel inclined to perform it.

Ulcers of the cornea constitute one of the most frequent complications of keratitis; it is, therefore, of great importance that the treatment should be thoroughly understood, and I should advise you always to keep in mind the principles I have just laid down. If you adopt them in your practice, you will scarcely ever see an ulcer followed by perforation of the cornea and loss of the eye.

*Perforation and fistulæ of the cornea—Hernia of the iris.*

Ulceration of the cornea sometimes leads, as I have already told you, to the perforation of that membrane; but this accident is most frequently observed in the purulent ophthalmia of new-born children, and in the Egyptian or gonorrhœal form of purulent ophthalmia. A portion of the cornea, nearly always situated in the centre, softens, assumes a yellow tint, and rising above the level of the surrounding parts, at last bursts. The perforation is sometimes sufficiently large to allow the crystalline lens to pass; when this is not the case, the tumours of the eye only escape.

It is a fact worthy of notice, that perforation of the cornea may be caused by inanition. M. Magendie found that it soon occurred in dogs, to which no nutriment was given, or that were fed on sugar. I have several times observed the same phenomena in patients who had been long deprived of aliment, or who had been exhausted by repeated blood-letting. In this case the symptoms which precede the perforation differ, in some respects, from those which are observed when it occurs in purulent ophthalmia. A circumscribed portion of the cornea gradually becomes opaque, then softens, for it can hardly be said to suppurate, and perforation takes place, without its having been preceded by any rising or swelling of the membrane. As the perforation may be small, and is not always situated opposite the pupil, it is not necessarily followed by loss of sight.

Every possible measure, calculated to prevent the occurrence of such an accident, must be adopted by the medical attendant, for when it has taken place, a palliative treatment only can be employed.

Owing to the firm and resisting nature of the cornea, a perforation of that membrane may remain open during a variable period, and thus deserve the name of a fistula; fistulous openings on the cornea are, however, extremely rare; I have only met with seven or eight instances during the entire course of my practice, and they are only observed when the perforation occupies the central portion of the membrane. Indeed, it is only in this region that the opening can, owing to the absence of the iris, remain free. One of the most remarkable cases of this kind that I have seen, was that of a young girl affected with hydrophthalmia. The cornea being greatly distended, paracentesis was performed, and the puncture remained fistulous during eighteen days. I also remember another case in which the perforation, caused by inanition, remained fistulous during three weeks.

Generally speaking, after perforation of the cornea, the iris being pushed forward, either simply closes the opening, or otherwise projects, so as to form a small tumour on the outer surface of the cornea. In some instances it is not the iris, but the vitreous humour which protrudes; and when this is the case, the tumour presents the appearance of a small transparent vesicle.

Various names have been given to the hernia, or prolapsus of the iris; thus it has been alternately denominated, *myocephalon*, *clavus*, *staphyloma of the iris*, &c. In the days of Galen, names were coined to represent the slightest peculiarity observed in a disease. The cause of this extreme diversity of nomenclature is partly to be found in the fact that medicine was then, in a great measure, in the hands of specialists, and when a man of powerful intellect is shut up in a small circle, his mind soon feels the want of aliment, and he, consequently, endeavours to extend the limits which are traced around him. Many plans of treatment are recommended against this affection, most of which are by no means so efficacious as those with whom they originated supposed them to be. Belladonna has been much lauded by some practitioners; as, however, it can only be useful when the prolapsus is quite recent, and when no adherence has yet been formed between the iris and the cornea, its utility becomes very restricted. The action of belladonna is purely mechanical; by dilating the membrane, it may, if the perforation be a certain distance from the circumference of the cornea, draw out of the opening that portion of the iris which protrudes. When the hernia of the iris passes the level of the cornea, it becomes a source of irritation to the eyelids, owing to the continual friction that takes place, and may occasion an exacerbation of the inflammation of the cornea, or its renewal, if it has been previously subdued. The exposed portion of the iris also often becomes the seat of great irritation, and gives rise to vegetations of various forms. When there is great inflammation existing, emollients and antiphlogistics should be employed; if, on the contrary, there be but little inflammation, the most efficacious plan of treatment which can be adopted is cauterization with the nitrate of silver: great care must be taken, in performing this slight operation, not to cauterize the cornea or the conjunctiva, as the inflammation would be thereby much increased. When the cauterization is properly executed, the patients generally feel great relief; but as it is extremely difficult to avoid injuring other parts of the eye, I would advise you only to resort to cauterization when it is indispensable. The astringent collyria usually employed in ophthalmiæ, may sometimes be used successfully. In one or two instances, in which I had prognosticated an aggravation of the malady, unless cauterization were resorted to, the patients got well in the course of a few months, although astringent collyria only had been employed. Since then I have, several times, allowed the disease to take its course, without attempting to cauterize, and the patients have likewise got well. You see, therefore, that we may occasionally temporize when the ophthalmia is slight, and there is no fear of suppuration and sloughing of the cornea. This, however, only applies to cases in which the perforation of the cornea is small; when it is large, and the prolapsus of the iris is considerable, cauterization must be resorted to as soon as the inflammation has subsided.—*London Medical Gazette.*

---

*On Poisoning by the Vapours of burning Charcoal and Coals.* By Golding Bird, M. D.—(Guy's Hospital Reports, No. viii.)—Dr. Bird considers it as doubtful, whether carbonic acid is the only deleterious agent produced during the combustion of carbonized substances. This appears to be countenanced by the fact mentioned by Sobernheim, that an apartment, in which individuals have been found asphyxiated, has been sprinkled with cream of lime, and cloths dipped in that fluid suspended in it, until every trace of carbonic acid has been absorbed; and yet persons, on entering such an apartment, have experienced the ordinary symptoms of poisoning by charcoal-vapour. Orfila ascertained that, from dimly burning charcoal, there was evolved carbonic acid, atmospheric air, nitrogen, and carburetted hydrogen; but when in a state of vivid combustion, only carbonic acid, common air, and nitrogen. The late Dr. Babington considered it probable, that the presence of carburetted hydrogen added greatly to the injurious effects of charcoal vapour, from the circumstance that the vapours arising from dimly burning charcoal are more injurious than those evolved during a state of brilliant combustion. Dr. Bird, however, thinks otherwise, as carburetted hydrogen is often present in very large proportion, in the atmosphere of coal mines, and yet the miners do not suffer any injury from breathing it. If charcoal be consumed in a confined space, not only is the air of the apartment deteriorated by the addition of the carbonic acid, but also by its having lost a portion of its oxygen, that portion which has united to the carbon to form the carbonic acid. Hence the air contains a larger proportion of nitrogen, and less of oxygen than common air.

It has not been found that a very large percentage of carbonic acid is required to produce fatal asphyxia. Small quantities, though at first not productive of any unpleasant feelings in persons inhaling it, will nevertheless cause serious and even fatal results in a space of time varying from a few minutes to some hours; indeed, in many of the recorded cases of fatal asphyxia, the persons who first entered the room to remove the body have not experienced any bad effects from their exposure to the noxious atmosphere.

Dr. Bird has recorded two instances of the alarming symptoms which may be produced from breathing an atmosphere vitiated by charcoal vapour. A gentleman retired to his study heated by one of Joyce's patent stoves. Within an hour he felt some pains in his head, accompanied by dizziness, which induced him to open the window when these disagreeable sensations quickly vanished. He closed the window and again sat down to his studies; but, at the end of an hour, he felt so seriously indisposed that he was compelled to leave the apartment. He had intense pain of the head, with a sense of constriction around the temples and forehead, resembling that produced by a tightly bound cord; his pupils were widely dilated, and contracted but feebly under the stimulus of light; he had singing in his ears; his pulse was 120, feeble, but regular; his face was very pale, and his lips livid; his extremeties were cold; his hands of a faint purple colour; his respiration was rather laborious and irregular; and the prostration of strength was so great, that he was unable to maintain the erect posture. By the abstraction of blood, and the administration of stimulants he recovered.

The other was of a more alarming nature. The Church of Downham, Norfolk, was heated by two of Joyce's patent stoves. For two Sundays they were used without any injurious effects, but as they heated the church insufficiently, care was taken to make the stoves consume the charcoal as completely as possible. In the middle of the morning service, a lady feeling some oppression, requested that a window might be opened. Soon afterwards some charity children were taken out of the church on account of their becoming affected. Mrs. O. was soon afterwards seized with headach and vertigo. Another lady was seized with similar symptoms, which she said she experienced on the two preceding Sundays. Immediately afterwards Mrs. B. was carried out of the church quite helpless; and another young lady followed her. Miss W. experienced a sense of constriction across the throat and around the head. She endeavored to leave the church, but found herself unable to maintain the erect position; she was therefore carried out, and was confined to her bed until the next day, with an intense throbbing headach. The clergyman, in consequence of these accidents, abruptly concluded the service. About seventy persons were more or less affected.

Two fatal cases of asphyxia produced by the inhalation of the noxious vapour evolved from burning charcoal are narrated.

James Trickey, aged 66, watchman and steeple-keeper of St. Michael's Church, was placed in the church in charge of a stove heated by charcoal, one of Harper and Joyce's patent apparatus. He entered the church, the first night it was tried at 11 o'clock P. M. on the 17th of November, 1838; and the next morning was found dead, lying on his face, with his feet about three feet from the stove. His head was lower than the rest of his body from the presence of a step elevating the trunk toward the stove. A considerable quantity of vomited food was found on the floor near his mouth. The church was so full of some vapour that the respiration of the persons who first entered in the morning were considerably affected.

The body was examined on the evening of the 18th. The countenance presented a remarkable appearance of placid and calm repose; the eyes were lustrous, and the pupils rather dilated.

*Head.*—On sawing through the calvarium, part of the membranes were lacerated, so that three or four ounces of bloody serum escaped. The vessels on the surface of the brain were gorged with blood. Considerable effusion of serum was found between the arachnoid and pia mater, as well as at the base of the brain; and on cutting into the substance of the brain, numerous bloody points presented themselves. The blood was florid in every part of the brain.

*Chest.*—The lungs were very dark, almost black, with the exception of the margins, which were red; the vessels were turgid with florid blood. The heart was healthy, and contained one or two small fibrinous clots. The trachea contained a quantity of frothy mucus; the lining membrane was injected, and of a bright red colour. The blood in the chest was fluid; that in the veins being of a very dark colour.

The abdominal viscera were healthy.

In order to ascertain the true cause of death in this case, the stove was charged with forty-nine pounds of charcoal, which were lighted, and the church shut up at 6 A. M. At 4 P. M. the doors were unlocked and the church entered. A very disagreeable sensation was experienced on entering the middle aisle, where the stove was placed, attended with difficulty of respiration. The air a few feet above the floor contained enough of carbonic acid gas to render lime water rapidly milky. Just below the step, where the poor fellow's head had been found lying, so much of the acid gas was present, that perfectly limpid lime-water being poured into a saucer scarcely reached it before it became perfectly opaque. Dr. Bird, who performed these experiments, was seized with palpitations of the heart, a sense of swimming in the head, of constriction across the temples, and an almost intolerable pain about the occiput; and he could scarcely maintain the erect position. One of the church wardens and the sexton were similarly affected. These results confirmed the opinion, that death was caused by the inhalation of carbonic acid gas.

George Bell, aged 38, on the 22d of November 1838, whilst clearing a lime-kiln, fell down insensible, after he had been about four minutes on the chalk, about two feet below the top of the kiln, and with his face downwards. His face was pale, his eyes glazed, and his pupils contracted. He was comatose, with stertorous breathing; his pulse was about fifty and feeble; and his skin was covered with perspiration. He was carried home in the erect posture, when violent tetanic spasms came on. Blood was drawn from the arm, blisters were applied, and stimulants administered. The spasms abated somewhat in severity, but the comatose state continued till the 25th, when he expired,—about eighty hours after his removal from the kiln.

His body was muscular, extremely rigid, and of a light purple colour posteriorly. Many small irregular purple blotches were on the anterior surface of the thighs, groins, and abdomen; and a few were on the chest and arms; the face was pale and the pupils dilated.

*Head.*—The vessels of the dura and pia mater as well as the sinuses were congested with blood. The basilar arteries and those of the corpus callosum were full of clotted blood. There was slight subarachnoid serous effusion; and about a drachm of fluid in the lateral ventricles. The cerebral substance presented many bloody points when cut into. About six drachms of serous effusion were found at the base of the brain.

*Chest.*—The lungs were slightly emphysematous, und scarcely at all collapsed. The mucous membrane at the bifurcation of the bronchi was slightly vascular, that of the larynx and trachea was natural. The mucous membrane of the pharynx was somewhat vascular. Some white froth was found in the trachea. The heart and pericardium were of a healty appearance. The blood in the larger vessels was coagulated and of a dark colour.

*Abdomen.*—The mucous membrane of the cœcum was of a deep purple hue as if echymosed and was of unusual thickness. The stomach, intestines, and other viscera were healthy.

Air taken from the mouth of this kiln was found to consist of carbonic acid 4, common air 67, and nitrogen 29 per cent.

Dr. Bird then gives a very full account of the symptoms caused by the inhalation of charcoal vapour, of the external appearance of the body, and of the appearances found on dissection, deduced from the cases related by the various authors who have written on this subject. He has also added two tables containing most of the published cases; in one of which all the varied appearances of the corpse, and in the other the appearances on dissection are noted. It is worth while to notice shortly the appearances, which may be regarded as pathognomonic of this variety of asphyxia, in order to prevent its being confounded with other causes of death.

The body is almost invariably studded with spots of a blueish or reddish brown or violet colour. These are most numerous on the most depending parts of the body. The eyes retain their lustrous aspect; and the pupils are invariably found dilated. The features present an appearance of calm repose, even slight distortion being extremely unfrequent. No reliance can be placed on the rigidity or flexibility of the limbs, or the slow cooling of the body, or the bloated or pale appearance of the face. The above are the only constant appearances.

On dissection, the blood-vessels and sinuses of the brain are invariably found turgid with blood; and serous effusion is met with beneath the arachnoid membrane, and within the ventricles of the brain. The blood may be florid or dark-coloured, fluid, or coagulated. Effusion of reddish serum into the pericardium and pleura is very frequent; but the lungs vary very much in appearance, having no fixed character. The left ventricle of the heart and the great blood-vessels are usually found empty. The mucous membrane of the larynx and trachea is often found reddened and covered with froth; sometimes, however, it is pale.

These are almost all the appearances which Dr. Bird regards as strictly pathognomonic of asphyxia from charcoal vapour. Various other diseased appearances are occasionally met with, but not with sufficient frequency to allow of any reliance being placed on them.—*Edinburgh Medical and Surgical Journal, Oct.* 1839.

---

*On Paraplegia depending on Disease of the Ligaments of the Spine.*—By C. Aston Key. (Guy's Hospital Reports, No. vi.)—As very few cases are on record where paraplegia has been traced to disease of the ligaments of the spinal column, unconnected with diseased bone, the following will be read with interest.

1. Samuel D——, aged forty-eight, of strong frame and healthy appearance, was admitted into the hospital on the 21st October, 1835. He had a stricture of the urethra, and a fistulous opening in the perineum. His urine, which was abundant and of natural appearance, chiefly passed through the perineal aperture. About the 8th of November, he complained of being unwell, and on the 10th, he became suddenly delirious, attended with feverish symptoms. These symptoms abated under the use of sinapisms, opium, and ammonia, and counter-irritation to the back of the neck. He continued improving till the 28th, but was found to have nearly lost the power of motion in the right leg. Fever returned, with affection of the sensorium, and a very diminished secretion of urine; and he complained of severe pain across the upper region of the abdomen, extending into each loin, especially the left. In the second week of December, the left leg became paralytic, and he continued incapable of moving either, though he imperfectly retained sensation in both. On the 25th, he was attacked with diarrhœa, all his other symptoms became aggravated, and he died on the 3d of January, when the following appearances were found on dissection.

The viscera of the chest, and those of the abdomen, with the exception of the colon, were healthy. The colon was inflamed throughout its inner surface, a large portion of which had been removed by ulceration. Over the right kidney was a peritoneal cell, which contained feculent matter, opening into the colon. The rectum, near the anus, presented several extensive and recent ulcers. Both kidneys were injected with blood; and in one were two or three minute points of suppuration; their structure was firm. The bladder was much thickened. In the substance of the psoas muscle a large abscess had formed, extending from near the origin of the muscle to its insertion, and communicating with a collection of purulent matter in the hip-joint, the *ligamentum teres* of which, and part of the articular cartilages, were destroyed by ulceration. The head of the femur and acetabulum seemed to be expanded; and the neck of the former was surrounded with bony deposits of old formation. The vertebræ were free from ulceration. The bodies of the lumbar vertebræ were covered with irregular prominences of bones, and the intervertebral substances projected more than usual. Within the canal of the spinal column the ligaments covering the intervertebral substance between the second and third lumbar vertebræ were found hardened and prominent, and projected so far into the canal as to diminish it by one-third of its diameter; thus causing considerable pressure on the spinal marrow.

2. George Weeks, aged forty, rather tall, and of a healthy appearance, has been for some years employed in carrying out beer for a public-house, which has exposed him to long-continued and fatiguing exercise. His health has been, in general, good, but twelve months ago he experienced a feeling of weakness in the left knee, which soon extended to the foot, accompanied with numbness and a sensation of cold. The right leg became similarly affected in a week or two, and he suffered from catching pains in the hip-joint on motion. He was obliged to leave off work from the increasing weakness of his legs a few days ago. Two months ago, he first noticed a want of power in the *sphincter ani*, which had increased so much in a month that he could with difficulty retain his motions. From the same period giddiness supervened upon any exertion.

On the 6th of July, 1836, the day of his admission to the hospital, he had almost entirely lost the power of motion in the lower extremities; and a sensation of tingling, numbness, and occasional pain, extended from the loins downwards; he was unable to empty his bladder, except in drops; and his command over the *sphincter ani* was very imperfect, his motions frequently passing involuntarily. He had no pain in his back, either on pressure or on motion; no pain in the head, only occasional giddiness. He had the free use of his arms, and his appetite was good, but his sexual passion was extinct.

His urine was strongly ammoniacal on the 25th July. Fœtid purulent matter flowed involuntarily from his bladder on the 6th August, and continued to come away at intervals, when he was raised to the erect posture, till his death; he had no command over the lower extremities, sphincter of the anus, or bladder. He died on the 22d August, when the following appearances were observed on dissection.

The brain was somewhat small, but appeared natural. The liver, spleen, and kidneys, did not appear healthy. A few points of purulent deposit were found in the kidneys. The bladder and ureters were extensively coated with sloughy fibrinous adhesive layers, and their surfaces were bathed with a bloody purulent secretion.

The intervertebral substance, about the twelfth dorsal vertebra, with the ligament covering it, presented a slight ridge, projecting into the medullary canal, as if an ossification from the edge of one bone tended to unite with a similar growth from the opposite edge. This transverse ridge manifestly narrowed the canal, as was very evident on passing the finger from the wider to the contracted part. The arches of the vertebræ and the medulla having been removed, no very visible displacement of the bones was perceived; but on making a vertical section in the median plane the fore outline of the spinal canal looked at this place as if the under bone had receded, whilst the front view of the bodies of the vertebræ still offered an unbroken line. It now appeared that the adjoining edges of bone, as well as the intervertebral substance, projected into the canal, the prominence of the last being the most considerable. The medulla was carefully examined, but seemed quite sound.

A number of similar cases are narrated, all tending to prove that paraplegia may sometimes depend on thickening of the ligaments of the spinal column; and Mr. Key is of opinion that "the ligaments are more frequently in fault than any of the structures of the spinal column, and that many of the cases of paralysis, whether temporary or permanent, and vaguely classed under functional derangements of the spinal cord, will be found, by dissection, to depend on an altered condition of these textures."—*Edinburgh Medical and Surgical Journal.*

